# New Cardiovascular Biomarkers in Ischemic Heart Disease—GDF-15, A Probable Predictor for Ejection Fraction

**DOI:** 10.3390/jcm8070924

**Published:** 2019-06-27

**Authors:** Daniel Dalos, Georg Spinka, Matthias Schneider, Bernhard Wernly, Vera Paar, Uta Hoppe, Brigitte Litschauer, Jeanette Strametz-Juranek, Michael Sponder

**Affiliations:** 1 Division of Cardiology, Medical University of Vienna, 1090 Vienna, Austria; 2 Division of Cardiology, Paracelsus Medical University of Salzburg, 5020 Salzburg, Austria; 3 Department of Pharmacology, Medical University of Vienna, 1090 Vienna, Austria

**Keywords:** heart failure, ejection fraction, soluble urokinase-type plasminogen activator receptor (suPAR), growth differentiation factor 15 (GDF-15), heart-type fatty acid-binding protein (H-FABP), soluble suppression of tumorigenicity 2 (sST2)

## Abstract

Background: Various biomarkers have been associated with coronary artery disease (CAD) and ischemic heart failure. The aim of this study was to investigate the correlation of serum levels of soluble urokinase-type plasminogen activator receptor (suPAR), growth differentiation factor 15 (GDF-15), heart-type fatty acid-binding protein (H-FABP), and soluble suppression of tumorigenicity 2 (sST2) with left ventricular ejection fraction (EF) in CAD patients and controls. Methods and Results: CAD patients were divided into three groups according to their EF as measured by the biplane Simpson method (53–84%, 31–52%, ≤30%). Overall, 361 subjects were analyzed. In total, 155 CAD patients had an EF of 53–84%, 71 patients had an EF of 31–52%, and 23 patients had an EF of ≤30% as compared to 112 healthy controls (age 51.3 ± 9.0 years, 44.6% female). Mean ages according to EF were 62.1 ± 10.9, 65.2 ± 10.1, and 66.6 ± 8.2 years, respectively, with females representing 29.0, 29.6, and 13.0%. suPAR, GDF-15, H-FABP, and sST2 values were significantly higher in CAD patients and showed an exponential increase with decreasing EF. In a multiple logistic regression model, GDF-15 (*p* = 0.009), and NT-brain natriuretic peptide (*p* = 0.003) were independently associated with EF. Conclusion: Biomarkers such as suPAR, GDF-15, H-FABP, and sST2 are increased in CAD patients, especially in highly impaired EF. Besides NT-proBNP as a well-known marker for risk prediction, GDF-15 may be an additional tool for diagnosis and clinical follow-up.

## 1. Introduction

Coronary artery disease (CAD) resulting in chronic heart failure (CHF) is still one of the most important topics in socio-economic fields despite various advancements in treatment options over the last decades.

Several pathophysiological processes such as inflammation or myocardial stress have to be considered to understand the complexity of CAD and the ventricular remodeling mechanisms leading to CHF. During the last years, cardiovascular biomarkers reflecting these sequences have raised the attention of many research groups in order to identify patients at risk at an early timepoint, as well as to optimize their treatment strategies.

Elevated levels of soluble urokinase-type plasminogen activator receptor (suPAR), a membrane-bound receptor, have been associated with coronary calcification [1], systemic inflammation [2], and CHF [3].

Growth differentiation factor 15 (GDF-15) is a transforming growth-factor beta cytokine that is mainly expressed in inflammatory settings [4,5], and its prognostic utility has been previously described in cardiovascular disease [6], especially in CHF [7].

Heart-type fatty acid-binding protein (H-FABP) represents a protein in cardiomyocyte cytoplasm and can be found in skeletal muscle [8], cardiac microvasculature, and endothelial cells as well [9]. H-FABP in cardiomyocytes is indispensable to ensure a high flux of long-chain fatty acids and therefore plays an important role in the energy supply.

Suppression of tumorigenicity (ST) 2 is an interleukin (IL)-1 receptor, which can occur as transmembrane receptor and a soluble form (sST2). The natural ligand of ST2 is IL-33, which acts as a traditional cytokine and as a transcription factor [10] binding to circulating sST2. Soluble ST2 serves as a decoy receptor inhibiting the IL-33/ST2-ligand (ST2L) complex resulting in an attenuation of IL-33-mediated inflammation [11], and consequently limits the cardioprotective effect of IL-33/ST2L activation. Increased levels of sST2 have been described in acute coronary syndromes [12], CHF [13], chronic obstructive pulmonary disease, and sepsis [14].

Although there is quite a wide range of studies depicting the impact of aforesaid biomarkers on poor clinical outcome in CHF [7,15,16,17,18,19,20], their detailed course of plasma levels in decreasing left ventricular ejection fraction (EF) has not yet been investigated. The aim of this analysis was to determine suPAR, GDF-15, H-FABP, and sST2 levels in CHF patients and, furthermore, to investigate their association with EF in comparison to the well-established biomarker N-terminal pro brain natriuretic peptide (NT-proBNP).

## 2. Methods

The clinical trials registration number was NCT02097199 (IPHAAB-study)/NCT02159235, under the title “Heavy Metals, Angiogenesis Factors and Osteopontin in Coronary Artery Disease”.

### 2.1. Subjects and Patient Population

Between October 2011 and December 2017, 249 patients with recently angiographically proven CAD were recruited in course of their inpatient stay at the Medical University of Vienna, Austria, in the Department of Internal Medicine II/Cardiology. Patients were included if coronary angiography revealed any atherosclerotic alteration in at least one coronary artery, regardless of any criteria necessitating coronary intervention. Patients were also included if any coronary intervention had been performed ≤7 days prior to enrolment. This cohort was further subdivided into three subgroups depending on left ventricular ejection fraction (EF): (1) 53–84%, (2) 31–52%, and (3) ≤30%. This classification was done in adherence to the current recommendations of the American Society of Echocardiography and the European Association of Cardiovascular Imaging [21] without any gender-specific analysis.

As a control group (to obtain reference values for the mentioned biomarkers) we recruited 112 subjects without angiographically proven CAD. In this group we performed a bicycle stress test (Ergometer eBike comfort, GE Medical Systems, Freiburg, Germany). Only subjects without corresponding anamnesis, typical symptoms, and/or ischemia-related ECG-abnormalities at rest or exhaustion were included in the control group.

In both groups detailed anthropometric and anamnestic data as well as routine laboratory parameters were assessed.

The study was carried out in adherence to the Declaration of Helsinki and its later amendments. The protocol has been approved by the Ethics Committee of the Medical University of Vienna and informed consent was obtained from all participating subjects prior to any study-related procedure.

### 2.2. Echocardiographic Analysis

Echocardiographic data was obtained with the use of the commercially available ultrasound systems (GE Medical Systems Vivid 7 Dimensions, Horton, Norway). All measurements were performed by experienced board-certified physicians according to the recommendations of the American Society of Echocardiography and the European Association of Cardiovascular Imaging [21]. The examiners were blinded to the levels of suPAR, GDF-15, H-FABP, and sST2. EF was calculated using the biplane method of disks with the following formula: (end-diastolic volume minus end-systolic volume) divided by end-diastolic volume (biplane Simpson method).

### 2.3. Laboratory Analysis

Blood samples were taken from an arm vein after 10 minutes in a lying position with a tube/adapter system. Samples were then placed in a standard centrifuge (Rotanta 460, Hettich GmbH & Co. KG, Tuttlingen, Germany) and were processed with 2500 rpm for 10 min. 

Serum levels of suPAR, GDF-15, H-FABP, and sST2 were analyzed by utilizing enzyme-linked immunosorbent assay (ELISA) kits that are commercially available (Duoset DY206, DY1678, DY807, DY957; R&D Systems, Minneapolis, MN, USA). Preparation of all necessary reagents and measurements were performed according to the instructions supplied by the manufacturer. In short, patient serum samples and standard protein were added to the wells of the ELISA plates (Nunc MaxiSorp flat-bottom 96-well plates, VWR International GmbH, Vienna, Austria) and were incubated for two hours. ELISA plates were then washed using a Tween 20/PBS solution (Sigma Aldrich, St. Louis, MO, USA). In the next step, a biotin-labelled antibody was added and plates were incubated for another two hours. Plates were then washed once more and a streptavidin-horseradish-peroxidase solution was added to the wells. By adding tetramethylbenzidine (TMB; Sigma Aldrich, St. Louis, MO, USA) a color reaction was generated. Optical density (OD) values were measured at 450 nm on an ELISA plate-reader (iMark Microplate Absorbance Reader, Bio-Rad Laboratories, Vienna, Austria).

The analysis was performed according to the manufacturer’s instructions. The coefficients of variation (CV) were for suPAR: 2.1–2.7% (intra-assay) and 5.1–5.9% (inter-assay); for H-FABP: 0.3–4.7% (intra-assay) and 1.3–17.4% (inter-assay); for sST2: 4.4–5.6% (intra-assay) and 5.4–7.1% (inter-assay); and for GDF-15: 4.7–5.9% (intra-assay) and 1.8–2.8% (inter-assay).

### 2.4. Statistical Analysis

Statistical analysis was done with SPSS 20.0 (IBM, Armonk, NY, USA). Dichotomous variables are expressed as frequencies or percentages. Continuous and normally distributed data is described by means ± standard deviation (SD), not normally distributed data as median/25th quartile/75th quartile. Comparisons between groups were made using the Chi-square or Fisher’s exact test for categorical variables, and the Student *t*-test or Mann–Whitney U test for continuous variables, as appropriate. Correlations between continuous parameters were calculated using the Spearman coefficient.

The influence of relevant parameters on EF was investigated first by univariate logistic regression. To identify the most relevant predictors a multiple regression model was selected from the scope of variables that reached statistical significance in univariate analysis by a backward procedure. The significance limit for a predictor to enter the model was 0.05. All tests were two-sided and *p*-values ≤ 0.05 were considered significant.

## 3. Results

### 3.1. Baseline Characteristics

Out of 249 CAD patients, 155 had an EF of 53–84%, 71 had 31–52%, and 23 patients had a severely impaired EF (≤30%). Patients were 62.1 ± 10.9, 65.2 ± 10.1 and 66.6 ± 8.2 years old, and were female in 29.0%, 29.6%, and 13.0% of cases, respectively. The control group consisted of 112 healthy adults with a mean age of 51.3 ± 9.0 years and with a higher proportion of women (44.6%). The detailed baseline characteristics concerning cardiovascular risk factors and routine laboratory assessment according to the four different groups are depicted in Table 1.

### 3.2. Biomarkers

**Figure 1:**
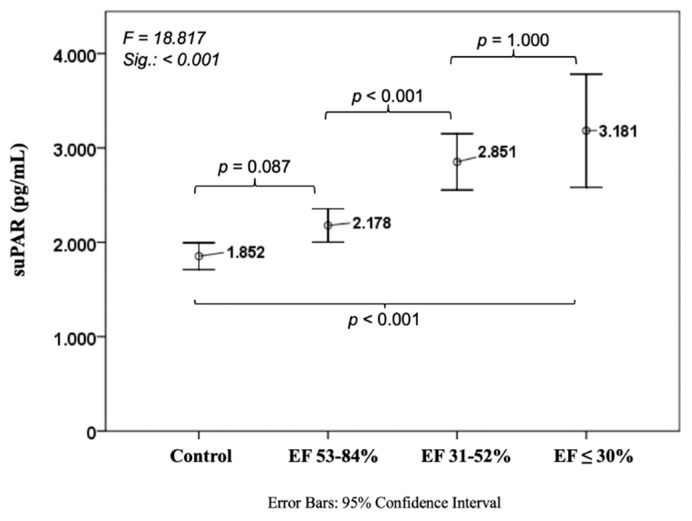
Course of soluble urokinase-type plasminogen activator receptor in ischemic heart disease. EF: ejection fraction; suPAR: soluble urokinase-type plasminogen activator receptor; EF: ejection fraction.

Every investigated biomarker showed a constant increase with decreasing EF. suPAR levels from controls were 1852 ± 759 pg/mL compared to 3181 ± 1387 in the worst EF group (*p* < 0.001). There was a slight trend between the control cohort and CAD patients with normal EF (1852 ± 759 vs. 2178 ± 1108 pg/mL, *p* = 0.087), whereas there was a steep increase between normal EF and EF 31–52% (2178 ± 1108 vs. 2851 ± 1260 pg/mL, *p* < 0.001) (Figure 1).

**Figure 2:**
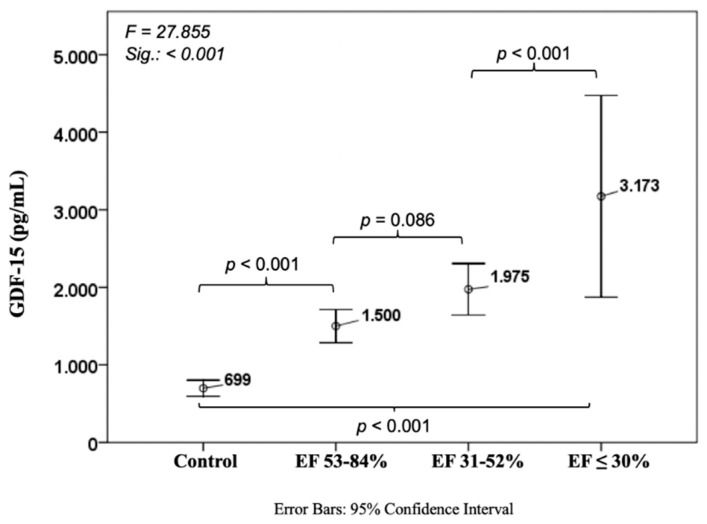
Course of growth differentiation factor 15 in ischemic heart disease. GDF-15: Growth differentiation factor 15.

Levels of GDF-15 were 699 ± 554 pg/mL in the control cohort with a significant difference to the group with EF ≤ 30% (3173 ± 3008, *p* < 0.001). We also observed a relevant distinction between controls and normal EF patients (1500 ± 1337, *p* < 0.001) and a slight trend between normal and mid-range EF (1975 ± 1405, *p* = 0.086) (Figure 2).

**Figure 3:**
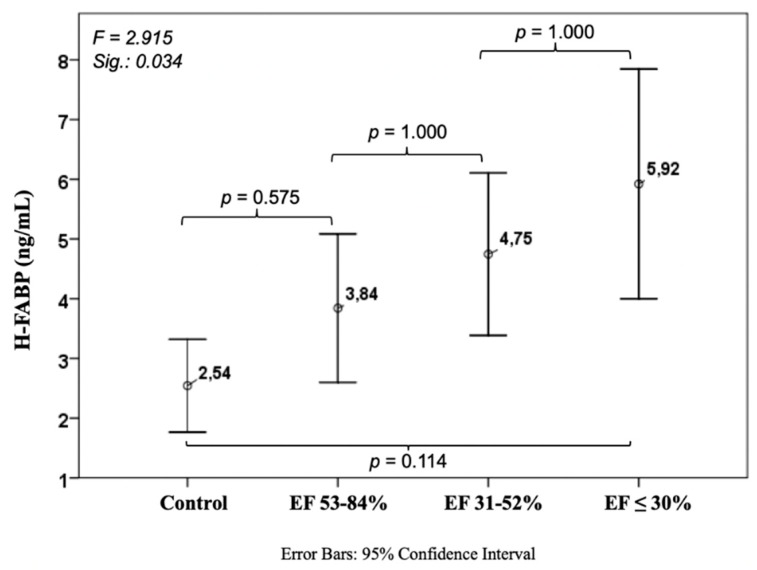
Course of heart-type fatty acid-binding protein in ischemic heart disease. H-FABP: heart-type fatty acid-binding protein.

Such as suPAR and GDF-15, H-FABP showed an increase with decreasing EF, but in contrast to the mentioned markers, this difference did neither show significant differences between controls 2.54 ± 4.16 ng/mL and EF ≤ 30% (5.92 ± 4.48 ng/mL, *p* = 0.114), nor in between the three EF groups (Figure 3).

**Figure 4:**
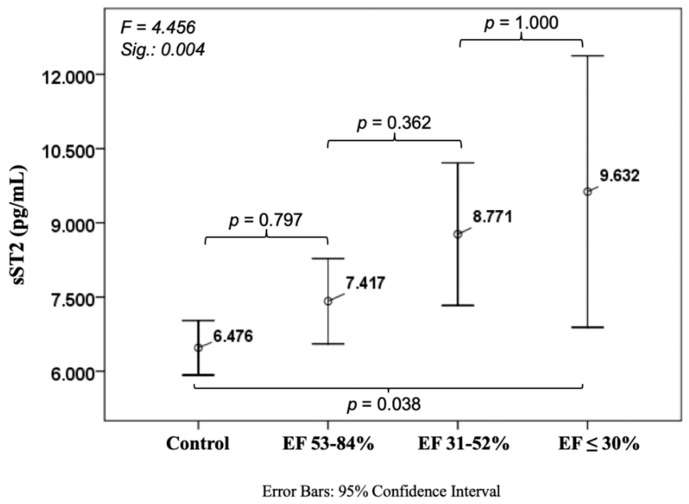
Course of soluble suppression of tumorigenicity 2 (sST2) in ischemic heart disease.

Soluble ST2 levels were 6476 ± 2916 pg/mL in patients without CAD and CHF with a considerable increase compared to the worst CHF patients (9632 ± 6346 pg/mL, *p* = 0.038). No differences were observed amongst the other cohorts (Figure 4).

**Figure 5:**
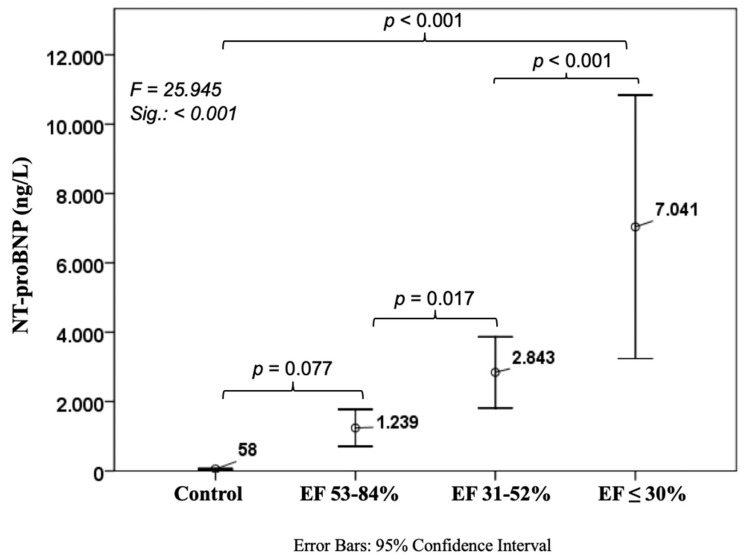
Course of N-terminal pro brain natriuretic peptide in ischemic heart disease. NT-proBNP: N-terminal pro brain natriuretic peptide.

Concerning the well-established NT-proBNP we found the expected values of 58 ± 103 ng/L in controls, with a significant rise in CHF patients with poor EF (7041 ± 8791 ng/L, *p* < 0.001). There was a trend between the control cohort and CAD patients with normal EF (1239 ± 3298 ng/L, *p* = 0.077), whereas there was a significant increase between normal and mid-range EF patients (2843 ± 4306 ng/L, *p* = 0.017) as well as between mid-range and poor EF (*p* < 0.001) (Figure 5).

In a multiple, backward logistic regression analysis, positive predictors for EF were age (*p* < 0.001), sex (*p* < 0.001), body mass index (*p* = 0.010), GDF-15 (*p* = 0.009) and NT-proBNP (*p* = 0.003, Table 2).

## 4. Discussion

In this analysis, we were able to confirm the diagnostic power of the biomarkers suPAR, GDF-15, H-FABP, sST2, and NT-proBNP in CHF patients compared to healthy adults. In addition, to the best of our knowledge, this is the first study to describe the detailed course of these biomarkers by means of left ventricular EF. Furthermore, we were able to show a significant association of GDF-15 with EF besides the well-established NT-proBNP in this specific patient population.

Circulating H-FABP levels increase during myocardial ischemia caused by pathological events, such as myocardial infarction, by leaking into the extracellular space [22]. However, in some cases myocardial damage due to acute physical activity might also increase its levels but they return to baseline within a few hours [23] and long-term physical training has been shown to lead to a significant decrease in H-FABP levels [24]. It can be assumed that high H-FABP levels in patients with low EF might be a sign of chronically impaired myocardial perfusion resulting in myocardial damage.

One could speculate that an increase in sST2 serum levels might be due to the chronic inflammatory state as it has been previously described in CHF patients [25]. As sST2 acts as a decoy receptor for IL-33, an upregulation might buffer an exuberant inflammation response.

Similar to sST2, suPAR (the soluble form of uPAR which is measurable after cleavage and release of membrane-bound uPAR) is involved in inflammation processes caused by numerous diseases, inter alia coronary calcification [1] and heart failure [3], and has been identified as marker for an unfavorable clinical outcome including mortality [26]. In our cohort we were able to confirm these findings as we found increasing suPAR levels dependent on the severity of CHF.

Interestingly, we observed an upregulation of GDF-15 with worsening EF as well as their independent association, together with NT-proBNP. GDF-15 is mainly secreted as a response to inflammation and hypoxemia. Due to the interaction with p53, it is released in sporadic severe stress situations, but also responds to low-level stressors during daily life activities [27,28]. These changes are reflected by circulating levels of GDF-15 that have been assigned protective effects regarding cell apoptosis [29] and myocardial hypertrophy [30,31].

While GDF-15 is expressed in visceral and subcutaneous adipose tissue in obese patients [32], it may also be released in atherosclerotic plaques in coronary arteries [33], in the myocardium in the course of acute cellular damages [5], as well as in peripheral tissue [34]. Due to its induction in different clinical scenarios, the use of GDF-15 as a diagnostic marker in acute cardiovascular care settings (e.g., chest pain, dyspnea) is limited.

In CAD, levels of GDF-15 are independently correlated with age, diabetes, high-sensitive C-reactive protein (hs-CRP), and natriuretic peptides in the AtheroGene study [35], which is in line with our finding of a significant correlation between GDF-15 and NT-proBNP in our CAD cohort (*r* = 0.727, *p* < 0.001). Furthermore, in the AtheroGene study, where 1352 patients with stable CAD were investigated, GDF-15 levels were independently associated with cardiac mortality. Schopfer et al. were able to depict the prognostic importance of GDF-15 concerning all-cause mortality in the Heart and Soul Study with 984 patients [36]. Circulating GDF-15 remains relatively stable after acute coronary syndromes without signs of HF, compared to other biomarkers such as cardiac troponin or hs-CRP showing a curve-shaped course [37,38], suggesting that GDF-15 reflects chronic disease burden.

In CHF with reduced EF, GDF-15 concentrations are elevated and their constant increase in relation to HF severity, as reflected by New York Heart Association functional class and NT-proBNP, has already been described [7,39]. This can be confirmed by our analysis with the additional input of a better characterized cohort by means of EF. Recently, the group of Li et al. investigated fewer patients with a much smaller control cohort than in our study and found similar results. The combination of GDF-15 and NT-proBNP significantly improved the accuracy of diagnosing HF [18]. Remarkably, circulating GDF-15 is also increased in HF patients with preserved ejection fraction (HFpEF) which is in line with the theory of Paulus and Tschöpe regarding a continuous inflammatory state as the central pathomechanism in HFpEF [40,41].

In severely impaired CHF patients that underwent implantation of left ventricular assist devices, the mechanical support has led to a significant decrease of measurable GDF-15, showing the reversibility of even highly increased levels [42]. How this reduction may affect the protective effects of GDF-15 remains a matter of debate, especially concerning potential therapeutic interventions targeting GDF-15 with unknown effects on clinical outcome.

In summary, elevated values of GDF-15 need to be interpreted with respect to other comorbidities, clinical symptoms and laboratory values. Likewise, with NT-proBNP values that may be adulterated by renal insufficiency or significant valvular disease, GDF-15 has to be evaluated simultaneously with other inflammatory parameters in order to rule out significant infections or even septic conditions.

## 5. Conclusions

Levels of the cardiovascular biomarkers suPAR, GDF-15, H-FABP, and sST2 constantly increase when left ventricular EF decreases in CHF patients, which is comparable to the course of the well-established NT-proBNP. However, only GDF-15 is significantly associated with EF in a multivariate model and therefore may expand the spectrum of biomarkers in identifying these patients without further needs of cost-intensive diagnostic modalities.

## 6. Limitations

Due to the single-center design of this study, a center-specific bias cannot be excluded and due to a small proportion of female patients we could not perform a sex-specific analysis. Additionally, unknown or un-controlled circumstances might have influenced the investigated parameters. Finally, although alterations in GDF-15 were shown in Caucasian and Asian tumor patients [43], there is little evidence regarding racial diversification in CAD and HF [44]. Our analysis was performed in Caucasian patients only; therefore, the results have to be interpreted cautiously with regard to other ethnicities.

## Figures and Tables

**Table 1:** Baseline characteristics.

	Control (*n* = 112)	EF 53–84% (*n* = 155)	EF 31–52% (*n* = 71)	EF ≤ 30% (*n* = 23)	*p*-Value
**Age (years)**	51.3 ± 9.0	62.1 ± 10.9	65.2 ± 10.1	66.6 ± 8.2	<0.001
**Female sex (%)**	44.6	29.0	29.6	13.0	0.006
**Hypertension (%)**	38.4	92.9	93.0	95.7	<0.001
**Family history of CAD (%)**	42.0	59.4	56.3	52.2	0.040
**Diabetes (%)**	5.4	21.9	25.2	34.8	<0.001
**Dyslipidemia (%)**	34.8	92.3	91.5	73.9	<0.001
**BMI (kg/m^2^)**	27.4 ± 4.2	27.8 ± 4.8	28.6 ± 5.7	29.5 ± 6.4	0.226
**SBP (mmHg)**	138 ± 16	131 ± 16	128 ± 16	129 ± 19	0.004
**DBP (mmHg)**	84 ± 12	76 ± 10	74 ± 10	79 ± 17	<0.001
**HR (bpm)**	67 ± 9	68 ± 12	71 ± 15	77 ± 13	0.003
**Cholesterol (mg/dL)**	200 ± 40	180 ± 52	164 ± 41	163 ± 56	<0.001
**Triglycerides (mg/dL)**	129 ± 78	153 ± 88	149 ± 83	148 ± 72	0.605
**LDL (mg/dL)**	117 ± 35	107 ± 40	94 ± 41	96 ± 35	0.495
**HDL (mg/dL)**	58 ± 18	48 ± 14	43 ± 12	37 ± 10	0.005
**Creatinine (mg/dL)**	0.9 ± 0.2	1.1 ± 0.8	1.2 ± 0.4	1.4 ± 0.5	0.003
**ASAT (U/L)**	26 ± 13	52 ± 61	52 ± 78	35 ± 25	0.311
**ALAT (U/L)**	27 ± 15	37 ± 27	43 ± 12	31 ± 25	0.569
**Gamma GT (U/L)**	30 ± 40	56 ± 100	89 ± 133	78 ± 88	0.076
**HbA_1_c (rel%)**	5.4 ± 0.6	6.0 ± 0.9	6.6 ± 1.7	7.1 ± 2.4	<0.001
**Erythrocytes (T/L)**	4.7 ± 0.5	4.5 ± 0.6	4.4 ± 0.6	4.6 ± 0.7	0.028
**Hemoglobin (g/dL)**	13.8 ± 1.4	13.3 ± 1.8	12.8 ± 1.8	12.8 ± 2.4	0.012
**Hematocrit (%)**	40 ± 3	39 ± 5	39 ± 5	39 ± 7	0.392
**Platelet count (G/L)**	243 ± 56	236 ± 74	250 ± 101	217 ± 89	0.040
**Leukocytes (G/L)**	6.6 ± 1.7	12.7 ± 2.0	8.0 ± 2.5	7.2 ± 2.1	0.494

Continuous variables are shown as mean ± standard deviation; ALAT: alanine aminotransferase; ASAT: aspartate aminotransferase; BMI: body mass index; CAD: coronary artery disease; DBP: diastolic blood pressure; EF: ejection fraction; GT: glutamyltransferase; HDL: high-density lipoprotein; HR: heart rate; LDL: low-density lipoprotein; SBP: systolic blood pressure.

**Table 2:** Multiple logistic regression analysis for ejection fraction.

Parameters	Regression Coefficient B	Standard Error	Beta	T	*p*-Value
**Constant**	−2.056	0.334		−6.149	<0.001
**Age (years)**	0.029	0.004	0.379	7888	<0.001
**Sex**	0.351	0.083	0.188	2.614	<0.001
**BMI (kg/m^2^)**	0.020	0.008	0.115	2.584	0.010
**GDF-15 (pg/mL)**	9.177 × 10^−5^	0.001	0.158	2.614	0.009
**NT-proBNP (pg/mL)**	3.702 × 10^−5^	0.001	0.172	2.983	0.003
*F = 38.0*; *p < 0.001*; *r^2^_adj_:0.346*					

BMI: body mass index; GDF-15: growth differentiation factor 15; NT-proBNP: N-terminal pro brain natriuretic peptide.
